# Alteration of erythrocyte membrane polyunsaturated fatty acids in preterm newborns with retinopathy of prematurity

**DOI:** 10.1038/s41598-019-44476-w

**Published:** 2019-05-28

**Authors:** Charlotte Pallot, Julie Mazzocco, Cyril Meillon, Denis S. Semama, Corinne Chantegret, Ninon Ternoy, Delphine Martin, Aurélie Donier, Stéphane Gregoire, Catherine P. Creuzot-Garcher, Alain M. Bron, Lionel Bretillon, Niyazi Acar

**Affiliations:** 1 University Hospital, Department of Ophthalmology, Dijon, F-21000 France; 2 Centre des Sciences du Goût et de l’Alimentation, AgroSup Dijon, CNRS, INRA, Université Bourgogne Franche-Comté, Eye and Nutrition Research Group, Dijon, F-21000 France; 3 University Hospital, Neonatal Intensive Care Unit, Dijon, F-21000 France

**Keywords:** Fatty acids, Retinopathy of prematurity

## Abstract

Extremely preterm infants are at high risk for retinopathy of prematurity (ROP), a potentially blinding disease characterized by abnormalities in retinal vascularization. Whereas animal studies revealed that n-3 polyunsaturated fatty acids (PUFAs) may be of benefit in preventing ROP, human studies conducted on preterm infants during the 1^st^ weeks of life showed no association between blood n-3 PUFA bioavailability and ROP incidence and/or severity, probably because of the influence of nutrition on the lipid status of infants. In the OmegaROP prospective cohort study, we characterized the erythrocyte concentrations of PUFAs in preterm infants aged less than 29 weeks gestational age (GA) without any nutritional influence. We show that GA is positively associated with the erythrocyte n-6 to n-3 PUFA ratio, and particularly with the ratio of arachidonic acid (AA) to docosahexaenoic acid (DHA), in infants with ROP. A time-dependent accumulation of AA at the expense of DHA seems to occur *in utero* in erythrocytes of preterm infants who will develop ROP, thus reinforcing previous data on the beneficial properties of DHA on this disease. In addition, preliminary data on maternal erythrocyte membrane lipid concentrations suggest modifications in placental transfer of fatty acids. Documenting the erythrocyte AA to DHA ratio at birth in larger cohorts might be useful to set up new prognostic factors for ROP.

## Introduction

The incomplete vascularization of the retina in preterm infants carries a risk of developing retinopathy of prematurity (ROP). ROP is characterized by neovascular complications that are the consequence of an imbalanced oxygen supply to the retina. ROP was first described in 1942 by Terry, who termed it “retrolental fibroplasia”, referring to the total retinal detachment observed in the most severe stages of ROP^1^.

Retinal vascularization begins *in utero* by the development of the embryonic vasculature that fully regresses before birth. Then an own vascular network develops through two complementary mechanisms, vasculogenesis and angiogenesis. Vasculogenesis begins at 6 weeks of embryonic life by the formation of four vascular arcades. Angiogenesis then takes place until the first 2 weeks after birth and corresponds to the emergence of vessels from these arcades. This late maturation makes infants born before the end of the third quarter of pregnancy at high risk of ROP. Although better knowledge of risk factors and improved screening and treatment have significantly decreased the risk of developing ROP, this disease remains the major cause of blindness in preterm infants^2^. The incidence of ROP in developed countries is highly variable and ranges from 6 to 34%^3,4^. Preterm birth (commonly before 32 weeks of gestational age (GA))^5^ and lower birth weight (usually below 1500 g)^6^ are independent risk factors of ROP, whereas high oxygen saturation is the major cause for developing the disease^7^. Other risk factors such as red blood cell transfusion^8^, use of erythropoietin (EPO)^9^, hyperglycemia^10^, and maternal anemia^11^ have also been identified.

Polyunsaturated fatty acids (PUFAs) from n-3 series have been shown to reduce by more than 50% the incidence of early preterm birth^12,13^. They also appear to be strong regulators of perinatal retinal vascular development. Indeed, n-3 PUFAs have been shown to prevent the vascular architecture defects observed in a mouse model of oxygen-induced retinopathy, an animal model of ROP^14–17^. Particularly, n-3 PUFAs modulate cellular pathways activated by IGF-1^18^ and endothelial cellular proliferation through VEGF signaling^19,20^, with IGF-1 and VEGF the cellular pathways implicated in the pathogenesis of ROP^21–24^. To our knowledge, few data is available on the lipid or PUFA status in preterm infants developing ROP. Martin *et al*. evaluated the postnatal evolution of the whole blood PUFA concentration in preterm newborns and showed strong modifications in the levels of the major n-6 and n-3 PUFAs arachidonic acid (AA) and docosahexaenoic acid (DHA), but without finding any association with the ROP phenotype^25^. In a longitudinal study, Löfqvist *et al*. showed that a lower area under the curve of serum AA during the 1st month of life is associated with a later diagnosis of ROP^26^. However, in these studies lipids were measured, at least in part, after enteral and parenteral feeding, and might then have been influenced by the oral or intravenous lipid supplements.

In the OmegaROP study, we wished to evaluate the prospective value of erythrocyte concentrations of n-3 and n-6 PUFAs for the development of ROP in preterm infants. We measured the individual concentrations of n-3 and n-6 PUFAs in red blood cells collected immediately after birth, in order to limit the interference with lipid nutritional intakes, and recorded the incidence of ROP.

## Results

### Characteristics of the population

The characteristics of the population are presented in Table 1. Fifty-eight preterm infants born before 29 weeks GA were included. Six infants died and the mortality rate was 11.5%. Blood samples were obtained from 52 infants at a median time of 12 h and a maximum time of 48 h after birth. The population included five sets of heterozygote twins. No difference was observed between the ROP and the no-ROP groups for sampling time, gender, use of EPO and cerebral hemorrhage. ROP was associated with significantly higher sepsis and red blood cell transfusion (*p* = 0.0233 and *p* = 0.0255, respectively), lower GA and birth weight (*p* = 0.0009 and *p* = 0.0014, respectively) and higher duration of mechanical ventilation (*p* = 0.0004). The mean follow-up for ROP screening was 11.3 ± 4.5 weeks of life. The mean number of screening examinations was 3.6 ± 2.0 per infant. The incidence of ROP was 51.9% with three cases with type 1 ROP (11.1%) and 24 type 2 ROP cases (88.9%). Type 1 ROP subjects underwent laser therapy in both eyes. No intravitreal injection of bevacizumab was used. Twenty-six ROP cases (96.3%) were observed in zone 2 and one ROP case (3.7%) was observed in zone 3. No ROP in zone 1 was observed. There was one case of ROP at stage 1 (3.7%), 19 cases at stage 2 (70.4%) and seven cases at stage 3 (25.9%). Four subjects were classified as a stage “plus” and three of them underwent laser treatment.

**Table 1:** Main characteristics of the population.

	Total population *n* = 52	ROP *n* = 27	No-ROP *n* = 25	*P*-value
Sampling time (h)	12 [12–24]	12 [12–21]	12 [12–30]	0.0569
Male	26 (50.0)	14 (51.8)	12 (48.0)	0.7813
Gestational age (weeks)	27.1 [26.2–27.7]	26.5 [25.5–27.1]	27.6 [27.1–28.4]	**0**.**0009**
Birth weight (g)	887 [797–1081]	815 [735–967]	1020 [870–1160]	**0**.**0014**
ROP	27 (51.9)	27 (100)	—	—
ROP treated	3 (5.8)	3 (11.1)	—	—
ROP detection (weeks)	8.2 [6.6–9.5]	8.2 [6.6–9.5]	—	—
Mechanical ventilation (days)	7.0 [1.0–14.0]	11.0 [5.5–16.5]	2.0 [1.0–7.0]	**0**.**0004**
Sepsis	23 (44.2)	16 (59.2)	7 (28.0)	**0**.**0233**
Erythropoietin use	33 (63.4)	18 (66.6)	15 (60.0)	0.6179
RBC transfusion	25 (48.0)	17(62.9)	8(32.0)	**0**.**0255**
Cerebral hemorrhage	25 (48.0)	13 (48.1)	12 (48.0)	0.9914

Continuous variables are expressed as median [IQR], categorical variables are expressed as No. (%).

ROP: retinopathy of prematurity; RBC: red blood cells.

*p*-values in bold indicate a statistically significant difference (*p* < 0.05).

### Fatty acid composition of erythrocytes

**Figure 1:**
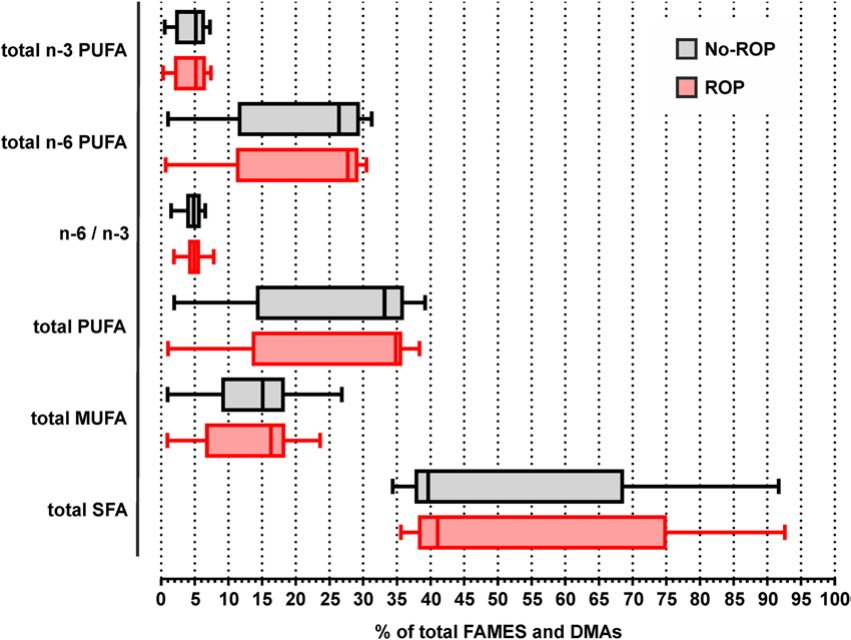
Distribution of the total n-6 to total n-3 PUFA ratio, total n-3 PUFA, total n-6 PUFA, total DMA, total MUFA, total PUFA and total SFA in the ROP and no-ROP groups. Results are presented as median, first and third quartiles, and range. ROP: retinopathy of prematurity; SFA: saturated fatty acids; MUFA: monounsaturated fatty acids; PUFA: polyunsaturated fatty acids; FAMEs: fatty acid methyl esters; DMAs: dimethylacetals.

No significant difference was observed between groups in individual fatty acids as well as in total saturated fatty acid (SFA), total monounsaturated fatty acid (MUFA), total PUFA, and total dimethylacetals (DMA) **(**Table 2**)**. The levels of total n-3 PUFAs, n-6 PUFAs and the total n-6 to total n-3 PUFA ratio were similar between the groups (*p* = 0.9513, *p* = 0.9120 and *p* = 0.8538, respectively). A striking characteristic of the measured erythrocyte fatty acid compositions of subjects from the ROP and no-ROP groups was high variability, especially in the levels of total SFA, total MUFA, total PUFA, total n-6 PUFA and total n-3 PUFA **(**Fig. 1**)**.

**Table 2:** Erythrocyte fatty acid composition of preterm infants without or with retinopathy of prematurity.

	No-ROP n = 25		ROP n = 27		*p* Value
	Median	IQR	Median	IQR	
C14:0	0.19	[0.10–0.23]	0.15	[0.09–0.20]	0.1631
C15:0	0.13	[0.10–0.24]	0.11	[0.10–0.18]	0.4062
DMA16:0	1.88	[1.49–2.31]	1.80	[1.54–2.08]	0.2302
C16:0	22.88	[20.76–33.63]	23.37	[21.29–30.04]	0.9581
C16:1n-9	0.27	[0.21–0.33]	0.23	[0.19–0.28]	0.4515
C16:1n-7	0.51	[0.37–0.56]	0.40	[0.23–0.51]	0.3892
C17:0	0.28	[0.24–0.64]	0.33	[0.25–0.72]	0.8881
DMA18:0	4.50	[4.08–4.87]	4.29	[3.91–4.77]	0.8615
DMA18:1n-9	0.82	[0.49–0.95]	0.93	[0.47–1.12]	0.4979
DMA18:1n-7	0.25	[0.19–0.36]	0.32	[0.18–0.40]	0.7582
C18:0	16.55	[14.63–28.33]	15.33	[14.82–30.25]	0.9437
C18:1t	0.15	[0.08–0.19]	0.17	[0.10–0.21]	0.8603
C18:1n-9	10.85	[7.99–12.84]	11.65	[6.36–12.96]	0.8250
C18:1n-7	2.38	[1.41–2.71]	2.45	[0.98–2.69]	0.8752
C18:2n-6 (LA)	3.24	[2.57–3.59]	3.04	[1.94–3.54]	0.8380
C20:0	0.29	[0.25–0.36]	0.29	[0.25–0.37]	0.4936
C20:1n-9	0.20	[0.17–0.22]	0.19	[0.16–0.25]	0.9558
C18:3n-3 (ALA)	0.06	[0.05–0.09]	0.08	[0.07–0.11]	0.4494
C20:2n-6	0.14	[0.12–0.16]	0.14	[0.14–0.16]	0.5954
C20:3n-9	0.57	[0.39–0.75]	0.48	[0.25–0.67]	0.4961
C22:0	0.52	[0.47–0.70]	0.46	[0.44–0.73]	0.9256
C20:3n-6	2.03	[1.58–2.59]	1.87	[1.44–2.22]	0.3053
C20:4n-6 (AA)	17.19	[7.98–18.96]	18.06	[8.78–19.54]	0.8558
C20:5n-3 (EPA)	0.54	[0.40–0.67]	0.52	[0.37–0.63]	0.4957
C24:0	1.51	[1.18–2.16]	1.23	[1.14–2.12]	0.9512
C24:1n-9	1.09	[0.96–1.35]	1.17	[0.98–1.34]	0.7550
C22:4n-6	2.26	[1.48–2.65]	2.45	[1.61–2.60]	0.7424
C22:5n-6 (n-6 DPA)	0.91	[0.43–0.98]	0.85	[0.55–1.12]	0.9322
C22:5n-3 (n-3 DPA)	0.37	[0.23–0.52]	0.45	[0.24–0.55]	0.4546
C22:6n-3 (DHA)	4.08	[1.71–4.86]	3.74	[2.11–4.96]	0.9276
Total SFA	39.68	[37.95–62.13]	41.08	[38.65–66.59]	0.9580
Total MUFA	15.08	[11.76–18.08]	16.31	[9.24–17.99]	0.8302
Total PUFA	33.20	[17.03–35.61]	34.86	[17.63–35.54]	0.9499
Total DMA	7.52	[6.71–8.07]	7.28	[6.71–8.06]	0.7101
Total n-3	5.16	[2.38–6.11]	5.16	[2.78–6.24]	0.9513
Total n-6	26.43	[14.19–29.06]	27.73	[14.58–28.88]	0.9120
n-6/n-3	4.85	[3.99–5.47]	4.91	[4.33–5.38]	0.8538

Results are expressed as % of total fatty acid methyl esters (FAMEs) + dimethylacetals (DMAs).

ROP: retinopathy of prematurity; IQR: inter quartile range; DMA: dimethylacetals; LA: linoleic acid, ALA; α-linolenic acid; AA: arachidonic acid; EPA: eicosapentaenoic acid; n-6 DPA: n-6 docosapentaenoic acid; n-3 DPA: n-3 docosapentaenoic acid; DHA: docosahexaenoic acid; SFA: saturated fatty acids; MUFA: monounsaturated fatty acids; PUFA: polyunsaturated fatty acids.

### Principal component analysis of erythrocyte fatty acid composition

**Figure 2:**
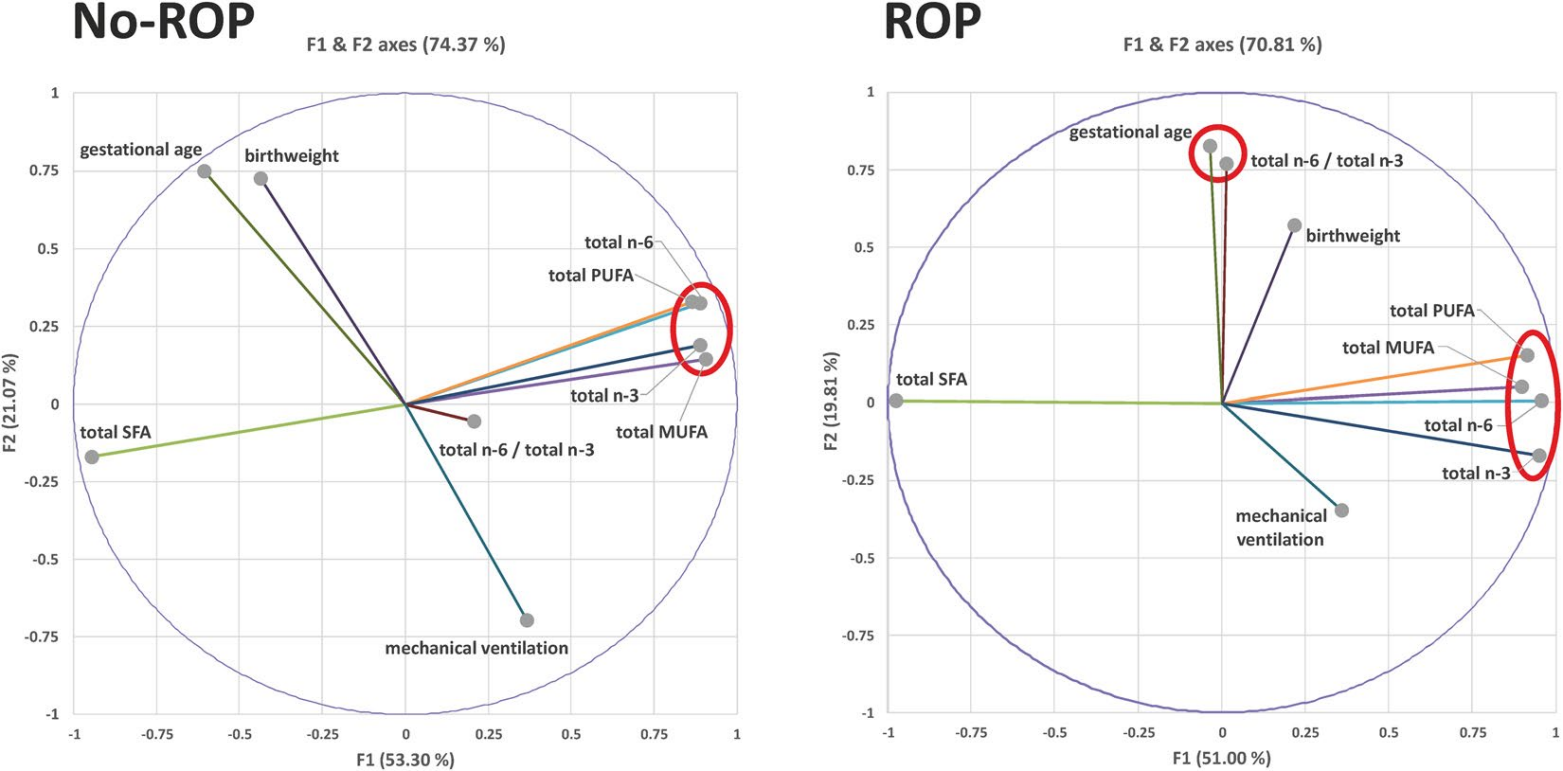
Principal component analysis of erythrocyte fatty acids and major risk factors of ROP in subjects with or without ROP. ROP: retinopathy of prematurity; SFA: saturated fatty acids; MUFA: monounsaturated fatty acids; PUFA: polyunsaturated fatty acids.

To better understand the origin of the data dispersion, we carried out a principal component analysis of the lipidomic data in relation to the major risk factors of ROP, namely mechanical ventilation, birthweight and GA **(**Fig. 2**)**. The F1- and F2-axes explained 53.30% and 21.07% of data variance in the no-ROP group and 51.00% and 19.81% in the ROP group, respectively. In the no-ROP group, data variance on the F1-axis was almost exclusively the refection of the variability in fatty acid composition (85.51% contribution to the F1-axis), whereas the risk factors for ROP were the major contributors of the F2-axis (83.75% contribution). In the no-ROP group, principal component analysis indicated that total MUFA, total PUFA, total n-6 PUFA and total n-3 PUFA formed a group loaded high on F1, whereas total SFA, birthweight and GA were very highly negative. The major difference in the ROP group when compared to the no-ROP group concerned the total n-6 to n-3 PUFA ratio, which switched from the center to a positive position on the F2-axis. This change resulted in the formation of a new cluster highly loaded on the F2-axis and composed of GA and the n-6 PUFA to n-3 PUFA ratio, suggesting a close interaction between these two variables in the ROP group.

### Associations between gestational age and erythrocyte lipids

**Figure 3:**
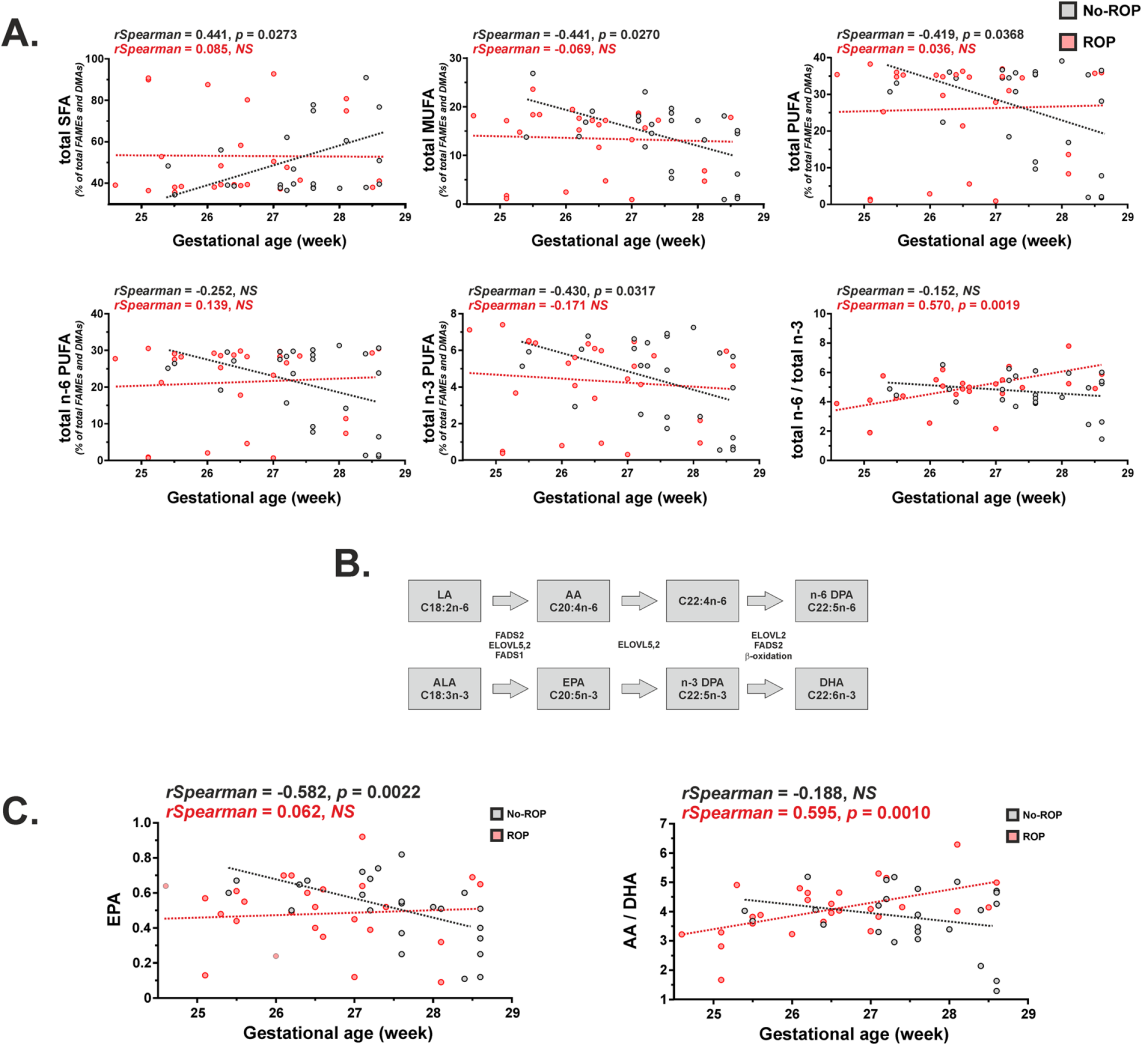
Origin of the differential evolution of the erythrocyte lipid composition according to gestational age in subjects with or without ROP. (**A**) Association between gestational age and total SFA, total MUFA, total PUFA, total n-6 PUFA, total n-3 PUFA and the total n-6 PUFA to total n-3 PUFA ratio in preterm infants with or without ROP. (**B**) Sequence of metabolic reactions leading to n-3 and n-6 long-chain PUFA synthesis. DHA and n-6 DPA are synthesized from ALA and LA, respectively, through metabolic reactions involving FADS2, ELOVL5,2, FADS1 and β-oxidation. (**C**) Association between gestational age and EPA, and the AA to DHA ratio in preterm infants with or without ROP. ROP: retinopathy of prematurity; SFA: saturated fatty acids; MUFA: monounsaturated fatty acids; PUFA: polyunsaturated fatty acids; FADS2: Fatty Acid Desaturase 2; ELOVL5,2: ELOngation of Very Long-chain fatty acid 5,2; FADS1: Fatty Acid Desaturase 1; LA: linoleic acid, AA: arachidonic acid; DPA: docosapentaenoic acid; ALA: α-linolenic acid; EPA: eicosapentaenoic acid; DHA: docosahexaenoic acid; NS: non significant; FAMEs: fatty acid methyl esters; DMAs: dimethylacetals.

Considering the results from principal component analysis, we checked for Spearman correlations between GA and total SFA, total MUFA, total PUFA, total n-6 PUFA, total n-3 PUFA and the n-6 PUFA to n-3 PUFA ratio **(**Fig. 3A**)**. In the no-ROP group, GA was positively associated with erythrocyte total SFA concentrations (*rSpearman* = 0.441, *p* = 0.0273) and negatively associated with erythrocyte total MUFA, total PUFA and total n-3 PUFA (*rSpearman* = −0.441, −0.419 and −0.430, respectively; *p* = 0.0270, 0.0368 and 0.0317, respectively). In the ROP group, only GA and the n-6 to n-3 ratio showed a significant positive association (*rSpearman* = 0.570, *p* = 0.0019).

We further investigated at which level of n-6 and n-3 PUFA metabolism the n-6 to n-3 ratio association with GA is discordant between the no-ROP and ROP groups. Long-chain PUFAs from the n-6 and n-3 series such as n-6 docosapentaenoic acid (n-6 DPA) and DHA are synthesized from linoleic acid (LA) and α-linolenic acid (ALA), respectively, through metabolic reactions involving desaturation by Fatty Acid Desaturase 2 (FADS2), elongation by ELOngation of Very Long-chain fatty acid 5,2 (ELOVL5,2) and Fatty Acid Desaturase 1 (FADS1) to synthesize AA and eicosapentaenoic acid (EPA), respectively, which are elongated by ELOVL5,2 to C22:4n-6 and n-3 docosapentaenoic acid (n-3 DPA), which in turn are elongated by ELOVL2, desaturated by FADS2 and finally β-oxidized **(**Fig. 3B**)**. Although no association was found between individual n-6 PUFAs and GA in the no-ROP and ROP groups, strong negative associations were found between EPA and GA in the no-ROP group (*rSpearman* = −0.582, *p* = 0.0022) and ALA and GA in the ROP group (*rSpearman* = −0.457, *p* = 0.0163) **(**Table 3 and Fig. 3C**)**. Within the different steps of n-6 and n-3 PUFA metabolism, only the ratio of most desaturated 22-carbon PUFAs, namely n-6 DPA and DHA, was positively associated with GA in the no-ROP group (*rSpearman* = 0.422, *p* = 0.0356). Interestingly, the ratio of the most prevalent PUFAs in the retina, AA and DHA, displayed a very strong positive association with GA in the ROP group only (*rSpearman* = 0.595, *p* = 0.0010) **(**Fig. 3C**)**.

**Table 3:** Spearman correlations between gestational age and erythrocyte n-6 and n-3 polyunsaturated fatty acids of preterm infants with or without retinopathy of prematurity.

	No-ROP *n* = 25		ROP *n* = 27	
	*rSpearman*	*P*-value	*rSpearman*	*P*-value
**n-6 PUFAs**				
C18:2n-6 (linoleic acid, LA)	−0.354	0.0817	0.230	0.2467
C20:4n-6 (arachidonic acid, AA)	−0.300	0.1447	−0.012	0.9511
C22:4n-6	−0.315	0.1251	0.026	0.8958
C22:5n-6 (n-6 docosapentaenoic acid, n-6 DPA)	−0.321	0.1169	−0.1233	0.5402
**n-3 PUFAs**				
C18:3n-3 (α-linolenic acid, ALA)	0.061	0.7693	−**0**.**457**	**0**.**0163**
C20:5n-3 (eicosapentaenoic acid, EPA)	−**0**.**582**	**0**.**0022**	0.062	0.7577
C22:5n-3 (n-3 docosapentaenoic acid, n-3 DPA)	−0.367	0.0712	0.009	0.9607
C22:6n-3 (docosahexaenoic acid, DHA)	−0.396	0.0500	−0.176	0.3774
**ratios**				
C18:2n-6/C18:3n-3 (LA/ALA)	−0.216	0.2978	0.3414	0.0813
C20:4n-6/C20:5n-3 (AA/EPA)	−0.202	0.3307	0.090	0.6540
C22:4n-6/C22:5n-3 (C22:4n-6/n-3 DPA)	−0.061	0.7710	0.251	0.2065
C22:5n-6/C22:6n-3 (n-6 DPA/DHA)	**0**.**422**	**0**.**0356**	−0.055	0.7837
C20:4n-6/C22:6n-3 (AA/DHA)	−0.188	0.3668	**0**.**595**	**0**.**0010**

ROP: retinopathy of prematurity; PUFA: polyunsaturated fatty acids; LA: linoleic acid, ALA; α-linolenic acid; n-6 DPA: AA: arachidonic acid; EPA: eicosapentaenoic acid; n-6 DPA: n-6 docosapentaenoic acid; n-3 DPA: n-3 docosapentaenoic acid; DHA: docosahexaenoic acid.

### Fatty acid composition of mother’s erythrocytes

**Figure 4:**
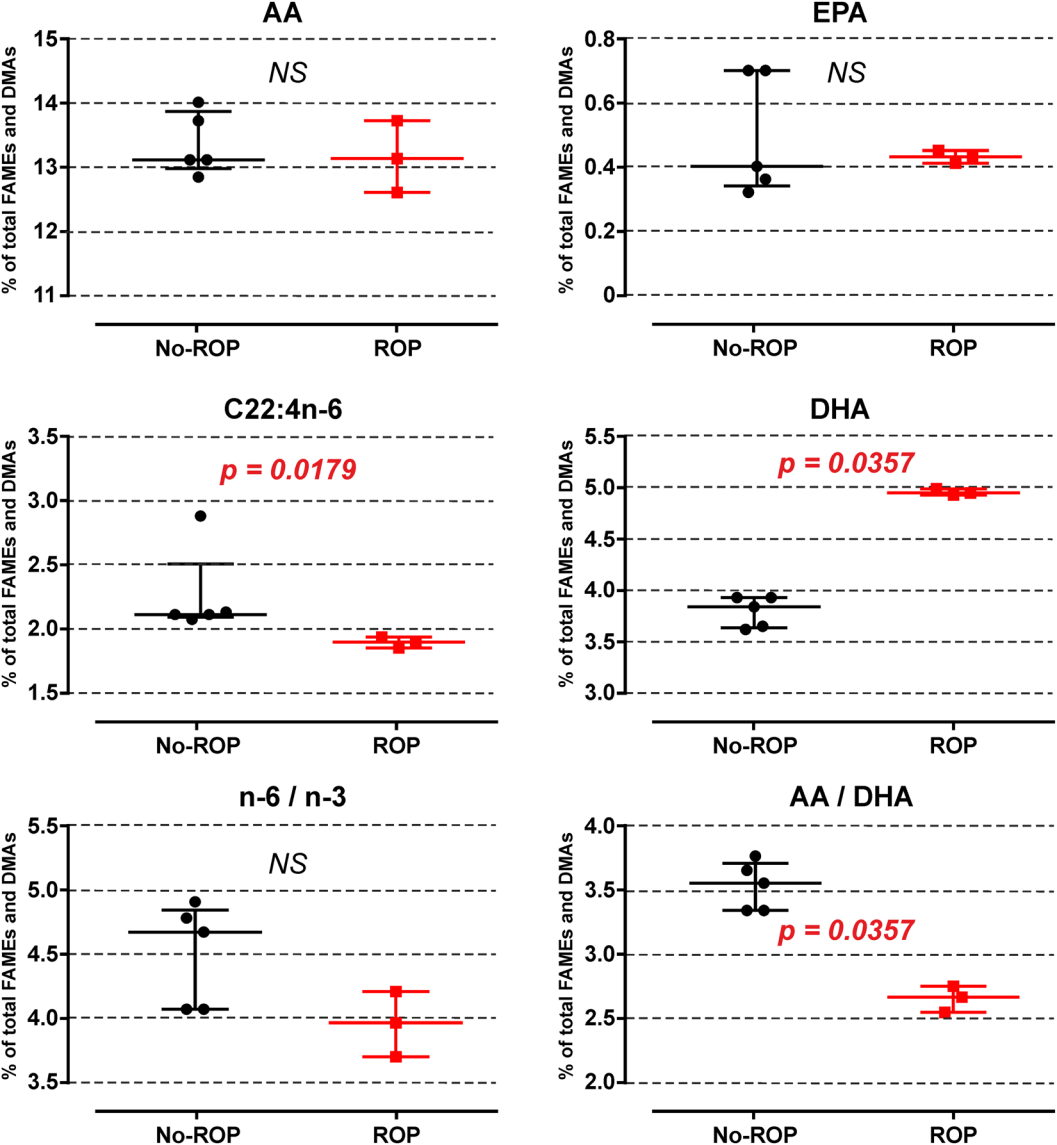
Erythrocyte membrane concentrations of selected PUFAs from n-6 and n-3 series in mothers of infants with or without ROP. Results are presented as median and interquartile range. ROP: retinopathy of prematurity; AA: arachidonic acid; EPA: eicosapentaenoic acid; DHA: docosahexaenoic acid; NS: non significant; FAMEs: fatty acid methyl esters; DMAs: dimethylacetals.

Eight mothers were included in the study. Within them, 3 gave birth to preterm infants that developed ROP. The fatty acid analyses showed a significant reduction in erythrocyte levels of C22:4n-6 (*p* = 0.0179) balanced by a significant increase of DHA concentrations (*p* = 0.0357) of mothers of preterm infants that will develop ROP **(**Fig. 4**)**. Whereas the n-6 PUFA to n-3 PUFA ratio was unchanged, mothers of children with ROP displayed a significantly reduced AA to DHA ratio in erythrocyte membranes (*p* = 0.0357).

## Discussion

This report characterizes the erythrocyte concentrations of fatty acids in preterm infants born before 29 weeks GA. The clinical characteristics of our population were comparable with those of several studies. The main risk factors for ROP in the present study were early GA, low birth weight and mechanical ventilation, as reported by others^11,27,28^. The incidence of ROP in our study was high (51.9%) and in agreement with other very-low-GA populations^29^.

Whereas Martin *et al*. and Löfqvist *et al*. looked at whole blood and plasma lipids, respectively^25,26^, the present study is to our knowledge the first characterizing erythrocyte lipids in preterm infants born before 29 weeks GA. The data show that the erythrocyte fatty acid profile in preterm infants born before 29 weeks GA resembles that measured in older subjects^30^. However, inter-individual variability was very high in our population. This might reflect the particular status of preterm infants and may suggest strong remodeling of fatty acid metabolism during gestation. This variability was not restricted to any specific fatty acid but seemed to concern all of them. The novelty of this study is that blood was sampled early in life in order to avoid any modification of the erythrocyte lipid status by the diet. Considering the erythrocyte lifetime ranging from 35 to 50 days in preterm infants^31^, and a blood sampling achieved within he first 48 h of life, the probability that the fatty acid compositions presented here are affected by the diet is null. Therefore, the variations observed in the present study might be assigned to differences in *in situ* fatty acid metabolisms rather than to a nutritional influence. In the studies reported by Collins *et al*. and Smither *et al*., the concentrations of total n-3 PUFAs were higher, but blood was sampled after 28 days of life^32,33^. Higher n-3 PUFA concentrations are therefore probably the consequence of the dietary supply during early life.

It is now well established that n-3 PUFAs play crucial roles in retinal angiogenesis and vascularization^14,34^. Whereas n-6 PUFAs display opposite properties, it was demonstrated that n-3 PUFAs limit the severity of pathologic retinal neovascularization and improve the regression of vascular lesions^14,16,34,35^. Epidemiologic studies have shown that a higher dietary consumption of n-3 PUFAs is associated with a decreased risk of neovascular forms of age-related macular degeneration (AMD)^36,37^, vascular abnormalities in diabetic retinopathy^38^ and vascular architecture defects in a mouse model of ROP^14–17^. The present study found no differences regarding the total erythrocyte levels of n-3 PUFAs between the ROP and the no-ROP groups, probably because of high inter-individual differences. The principal component analysis revealed a differential clustering of the n-6 PUFA to n-3 PUFA ratio with GA in ROP group, suggesting that GA is an important variable to consider when evaluating the levels of n-6 and n-3 PUFAs in preterm infants. We also observed that GA is positively associated with the n-6 PUFA to n-3 PUFA ratio in infants who will develop ROP, which can be the sign of a possible remodeling of the n-6 to n-3 balance in favor of n-6 PUFA series. Interestingly, and when regarding individual n-6 and n-3 PUFAs, only the AA to DHA ratio revealed a time-dependent accumulation of AA at the expense of DHA in erythrocytes in the ROP group. Since AA and DHA are precursors of metabolically active molecules involved in inflammation^39^, such a differential age-dependent evolution of the AA to DHA ratio can have consequences on the inflammatory status of infants, which is a known additional risk factor of ROP^40,41^. Moreover, n-3 PUFAs are involved in retinal vascularization by limiting the development of avascular areas and neovascularization processes at the early and late phases of the disease, respectively^14,34,35^. In these animal studies, n-6 PUFAs were not shown to be protective against the development of ROP.

Numerous human studies suggest that PUFAs from the n-3 series reduce the likelihood of premature birth^12,13^. Particularly, EPA and its oxygenated derivative Resolvin E3 have been shown to lower preterm delivery in mice^42^. A recent study on African Americans showed that a higher maternal erythrocyte membrane AA to DHA ratio was associated to an increased risk of preterm birth^43^. In a Japanese population, the length of gestational period positively correlated with DHA concentrations in maternal erythrocyte membranes^44^. DHA levels in erythrocytes was shown to range between 5.5 and 5.8% of total fatty acids in mothers who delivered nearly full-term and full term infants (38 to 40 weeks GA)^44,45^, and was shown to be reduced to until 2.5% of total fatty acids in mothers of 33 weeks-GA preterm infants^43^. Although they have to be confirmed at a larger scale, the data we obtained on mothers confirm these findings as we measured DHA at a level of 3.8% of total fatty acids in erythrocytes of mothers of preterm infants of less than 29 weeks GA. These findings reinforce the idea of supplementing mothers with n-3 PUFAs in order to reach blood levels consistent with an increased likelihood of delivering a full-term child. As one of the best way to reduce the incidence of ROP is to have a full-term delivery, it would consist in using the mother as a source of increased n-3 fatty acid delivery to the developing fetus.

Contrary to our data on infant erythrocyte membrane fatty acid composition, data obtained by Löfqvist and collaborators show that a reduced area under the curve of AA during the 1st month of life is associated with a higher risk of ROP^26^. These findings are somewhat unexpected regarding the numerous and consistent experimental data on the properties of n-6 and n-3 PUFAs. They are likely to be the consequence of the dietary supply of the newborns and underline the difficulty of drawing a reliable picture of blood fatty acid status in newborns over the 1st weeks and months of life.

In the present study, we can hypothesize that the differences observed between subjects who will suffer or not suffer from ROP might be the result of differential *in utero* bioavailability of n-6 and n-3 PUFAs. Indeed, it was shown that the AA and DHA balance of the fetus is regulated through the placental transfer of fatty acids from the mother^46^. Other findings have shown that whereas the AA to DHA ratio is initially high in the fetus, the maternal transfer of DHA increases during the last trimester of gestation, resulting in a lower AA to DHA ratio at term^47^. Our data are concordant with abnormalities in placental PUFA transfer in preterm infants who will develop ROP. In this study, we could also analyze erythrocyte fatty acids on a reduced number of mothers. These preliminary data seem to sustain such a hypothesis since they highlight differences in red blood cell concentrations of DHA between mothers of infants that will develop or not ROP. Particularly, we have found DHA levels in maternal erythrocytes to be of about 4.9% of total fatty acids, which appears to be high considering the length of gestation as shown by others and as discussed above^43–45^. Such a finding can be the consequence of DHA accumulation in mothers of infants who will develop ROP and can explain why the AA to DHA ratio in infants with ROP is increased with higher GA. However, these analyses have to be repeated on a larger number of mothers to confirm this hypothesis. Moreover, mechanistic studies are needed to determine whether defects in placental transfer of n-3 PUFAs are involved in the modifications in maternal and fetal erythrocyte fatty acids observed in the ROP group.

We acknowledge several limitations to our study. First, the number of subjects is low. Second, the population includes only three ROP cases that required treatment, whereas such cases are the most concerned by neovascular abnormalities.

In conclusion, this study shows that erythrocyte lipids of preterm infants who will develop or not develop ROP display different patterns in terms of n-6 and n-3 PUFA compositions. Since erythrocyte membranes are a good mirror of retinal lipid composition in the newborn^48–50^, these data raise the question of potential modifications of retinal lipid metabolism in ROP with potential links with inflammatory and angiogenic processes.

## Materials and Methods

### Ethics statement

Samples were collected in accordance with the guidelines of the Declaration of Helsinki. The experimental protocol was approved by local ethics committee (CPP Est III, School of Medicine, Dijon, France) that waived the need to obtain written consent. However, an information note was given to parents and/or legal guardians. In accordance with “ethical considerations for clinical trials on medicinal products conducted with the paediatric population”, the volume of blood collected was limited to 0.5 mL^51^.

### Selection of the patients

From July 31^st^, 2015, to January 31^st^, 2018, all preterm infants born before 29 weeks GA and hospitalized in the neonatal intensive care unit of the Dijon University Hospital, Dijon, France, were included in the study. A 0.5-mL blood sample was collected by venipuncture within the first 48 h of life in a heparinized tube. A 5-mL blood sample was also taken from a reduced number of mothers within a maximum delay of 5 days following child delivery. Red blood cells were immediately separated from serum and samples were stored at −80 °C until lipidomic analyses.

ROP screening was performed with the wide-field RETCAM II® camera (Clarity Medical Systems; Pleasanton, CA, USA) using a lid speculum after topical anesthesia by chlorhydrate oxybuprocain (1.6 mg/0.4 mL). Pupillary dilation was previously performed using one drop of 2.5% epinephrine (Phenylephrine 5% diluted to 2.5%) and one drop of tropicamide (2 mg/0.4 mL). The procedure was completed by a trained nurse and all fundus photographs were analyzed by a trained pediatrics-specialized ophthalmologist. Screening began at 4–6 weeks of life but never before 31 weeks of postconceptional age (PCA), and was repeated every other week until 39 weeks PCA if no ROP was detected, and every week and up to twice a week in case of ROP. ROP staging was determined according to the International Classification of ROP^52^. Patients were classified in the group suffering from ROP (ROP group) or in the group of unaffected controls (no-ROP group). Within the ROP group, subjects were classified into ROP type 1 and ROP type 2. The major risks of developing ROP, namely term and weight at birth, duration of mechanical ventilation, sepsis, use of erythropoietin, red blood cell transfusion and cerebral hemorrhage were documented.

### Lipidomic analyses

The red blood cell fatty acid composition was determined according to previously described procedures^53–55^. Briefly, total lipids were extracted from erythrocytes according to Moilanen and Nikkari^56^. Phospholipids were purified from total lipid extracts using silica cartridges^57^, and transmethylated using boron trifluoride in methanol^58^. The fatty acid methyl esters (FAMEs) and dimethylacetals (DMAs) formed were analyzed on a Hewlett Packard Model 5890 gas chromatograph as previously described^53–55^. The data were processed using the EZChrom Elite software (Agilent Technologies, Massy, France) and reported as a percentage of the total FAMEs and DMAs.

### Statistical analysis

Statistical analysis was performed using GraphPad Prism v6.05 (GraphPad Software, San Diego, CA, USA) and XLSTAT v2018.02.50494 (Addinsoft, Paris, France). Quantitative data are expressed as median and interquartile range [IQR]. The groups were compared using the nonparametric Mann-Whitney test for quantitative variables and the Chi-2 test or Fisher exact test for qualitative variables. A principal component analysis and Spearman correlations were used to analyze the extent to which the fatty acid composition of erythrocytes was associated with the major risk factors of ROP, namely mechanical ventilation, birthweight and GA. Linear regression analyses were carried out to compare the levels of individual or total n-3 and n-6 PUFAs or n-3 and n-6 PUFAs ratios as a function of GA. A *p*-value lower than 0.05 was considered as statistically significant and the tests were two-tailed.
